# Pocket depth and bleeding on probing and their associations with dental, lifestyle, socioeconomic and blood variables: a cross-sectional, multicenter feasibility study of the German National Cohort

**DOI:** 10.1186/1472-6831-15-7

**Published:** 2015-01-21

**Authors:** Heiko Zimmermann, Daniel Hagenfeld, Katja Diercke, Nihad El-Sayed, Julia Fricke, Karin Halina Greiser, Jan Kühnisch, Jakob Linseisen, Christa Meisinger, Nicole Pischon, Tobias Pischon, Stefanie Samietz, Marc Schmitter, Astrid Steinbrecher, Ti-Sun Kim, Heiko Becher

**Affiliations:** Institute of Public Health, University of Heidelberg, lm Neuenheimer Feld 324, 69120 Heidelberg, Germany; Department of Conservative Dentistry, Section of Periodontology, University of Heidelberg, lm Neuenheimer Feld 400, 69120 Heidelberg, Germany; Policlinic of Periodontology, University-Hospital of Münster, Münster, Germany; Department of Orthodontics, University of Heidelberg, lm Neuenheimer Feld 400, 69120 Heidelberg, Germany; Division of Cancer Epidemiology, German Cancer Research Center, Im Neuenheimer Feld, 581, 69120 Heidelberg, Germany; Institute for Social Medicine, Epidemiology and Health Economics, Charité-Universitätsmedizin Berlin, Luisenstr. 57, 10117 Berlin, Germany; Department of Conservative Dentistry and Periodontology, Ludwig-Maximilians-University of Munich, Goethestraße 70, 80336 München, Germany; Helmholtz Zentrum München, German Research Center for Environmental Health, Institute of Epidemiology II, Neuherberg, Germany; Department of Periodontology and Synoptic Dentistry, Charité - University Medicine Berlin, Berlin, Germany; Molecular Epidemiology Research Group, Max Delbrück Center for Molecular Medicine (MDC) Berlin-Buch, Robert-Rössle-Strasse 10, 13125 Berlin, Germany; Poliklinik für zahnärztliche Prothetik und Medizinische Werkstoffkunde Zentrum für Zahn, Mund- und Kieferheilkunde Universitätsmedizin Greifswald, Greifswald, Germany; Department of Prosthodontics, lm Neuenheimer Feld 400, 69120 Heidelberg, Germany; Institute of Medical Biometry and Epidemiology, University Medical Center Hamburg-Eppendorf, Martinistr. 52, 20246 Hamburg, Germany

**Keywords:** Periodontitis, Dental examination, BMI, Laboratory parameters, Bleeding on probing, German National Cohort

## Abstract

**Background:**

To investigate the periodontal disease status in a multi-center cross-sectional study in Germany. Associations of dental, socio-economic, blood and biomedical variables with periodontal outcome parameters were evaluated.

**Methods:**

From 4 different centers N = 311 persons were included, drawn randomly from the registration offices. Maximal pocket depth (PD) was used as primary indicator for periodontitis. It was classified as: no/mild ≤3 mm, moderate 4-5 mm, severe ≥6 mm. Associations between socioeconomic (household income, education), lifestyle, and biomedical factors and PD or bleeding on probing (BOP) per site (“Yes”/”No”) was analyzed with logistic regression analysis.

**Results:**

Mean age of subjects was 46.4 (range 20–77) years. A significantly higher risk of deeper pockets for smokers (OR = 2.4, current vs. never smoker) or persons with higher BMI (OR = 1.6, BMI increase by 5) was found. Severity of periodontitis was significantly associated with caries lesions (p = 0.01), bridges (p < .0001), crowns (p < .0001), leukocytes (p = 0.04), HbA1c (p < .0001) and MCV (p = 0.04). PD was positively correlated with BOP. No significant associations with BOP were found in regression analysis.

**Conclusions:**

Earlier findings for BMI and smoking with severity of PD were confirmed. Dental variables might be influenced by potential confounding factors e.g. dental hygiene. For blood parameters interactions with unknown systemic diseases may exist.

## Background

Periodontitis is a chronic infectious disease which results in biofilm formation on tooth and root surfaces and subsequent destruction of periodontal tissue because of an accelerated host response to pathogenic bacteria. Periodontitis is mostly promoted by microorganisms which are the primary cause for developing periodontitis [1, 2]. Severity and progression of periodontitis is linked to the microbiological burden, the susceptibility of the host and modified by environmental and behavioral factors [3]. Earlier studies found smoking [4, 5], diabetes mellitus [6–8] or other risk indicators, e.g. genetic predisposition [9–11], age [12, 13], sex [14, 15], social and psychological factors [15–17] to be correlated with periodontitis. There is evidence that periodontitis is a risk factor for other diseases. It might lead to higher risk for ischemic [18, 19] and hemorrhagic stroke [20], cardiovascular disease [21, 22], myocardial infarction [23, 24] and systemic diseases [25, 26].

Several studies have shown that periodontitis is highly prevalent in both developing and industrialized countries [27–30]. Recent research has also shown, that periodontitis is highly prevalent in Germany, especially in older ages [31]. In about 10% of periodontitis cases treatment is necessary even in young patients [32]. Due to the decrease in caries prevalence and the increased amount of maintained teeth periodontal infections are rising and are expected to even increase in future [31].

The prevalence of severe periodontitis (≥6 mm) in adult populations is 5-20% worldwide [30]. However, disease progression varies regarding different demographic characteristics including age, sex and behavioral factors like smoking [33]. To assess the burden of periodontitis [30] in Germany some surveys have been performed [34, 31, 35]. In Germany, 18% of individuals show a diagnosis of severe periodontitis in all age groups jointly [36].

The forthcoming German National Cohort (GNC) Study aims at recruiting a representative sample from the general population in Germany [37]. Recruitment will take place in 18 study centers distributed throughout Germany and will include 200.000 people aged between 20 and 69 years. The study aim is to investigate the development of major chronic diseases, subclinical disease stages and functional changes, and to identify strategies for prevention, prediction, and early detection of diseases.

Feasibility studies were conducted in all centers in 2012 to test specific aspects of the GNC. One of these aspects was related to oral health and to test the reliability of the clinical measurements performed by a study nurse compared to the measurement of a dentist and to test the required time for the assessment of the oral variables. Data on periodontal status of study participants in different regions and a wide range of lifestyle and health related variables were collected.

Based on these data we present in this paper results on pocket depth and bleeding on probing status as outcome parameters for periodontal disease and to quantify factors which are associated with these conditions.

## Methods

### Study population

Subjects participated in the pretest phase of a population-based interdisciplinary epidemiologic cohort study called “The German National Cohort” (GNC) [37, 38]. For more information see also http://www.nationale-kohorte.de/. Several feasibility studies have been performed in 2012 to test certain aspects of the main study. This study was performed in four of the 18 participating centers, Heidelberg (south-west Germany), Augsburg (south), Berlin and Greifswald (north-east Germany). These centers were selected because of their specific expertise to perform this particular study.

From the local registration offices random population samples were drawn. However, the feasibility studies were not designed to determine response rates, but to establish processes and to test methods for the final German National cohort in case of the basic program and the additional oral examinations. Some individual data on recruitment processes and response rates for each of the four centers are presented in the Table 1. Between the centers recruitment process (and numbers) varied.

**Table 1:** **Recruitment characteristics of the study population for the four centers**

Study center	Overall sample	Contacted	Subjects participated	Overall response P13 program	Drop-outs*	Recruitment: Additional approach	Persons for paper analysis
**Augsburg**	N = 920	N = 750 for P13	ROS**: N = 228	24.8% (228/920)	N = 522		N = 44
**Greifswald (Neu-Brandenburg)**	N = 291	N = 291 for P13	ROS: N = 148	50.9% (148/291)	N = 143	a.) Phone calls (30%), b.) House visits and media campains 70%	N = 107
**Berlin**	N = 1667	N = 1667 1. (N = 967 for P13, 2. N = 700, Basic program only)	ROS: N = 13 CS***: N = 63	7.9% (76/967)	N = 891	a.) ROS	N = 70
						b.) CS	
**Heidelberg**	N = 600	600	ROS: N = 143 of 600 (23.83%)	16% (96/600)	N = 75	a.) Phone calls 40.3% (242/600)	N = 90
						b.) Contacted without phone number (358/600)	

*Drop outs (unknown addresses, deceased or verbal communication problems, occupational reasons, refused…) **ROS Registration Office Sample ***CS Convenience Sample.

The joint overall sample finally incorporated n = 311 Germans (120 males, 191 females). The study approval was approved by the following ethics committees; (Charité - Universitätsmedizin Berlin (EA1/101/11), Medizinische Fakultät der Universität Heidelberg (S-108/2011), Medizinische Fakultät der Universität Greifswald (BB 12/11), Bayerische Landesärztekammer, München). All participants gave written informed consent. Individuals were invited by letter, with a written reminder and, if required, additional contacts by phone. Each person was interviewed and underwent a full program of medical examinations which includes a blood sample and the oral examination reported in this paper. The total examination time for each person was about 2.5 hours. The dental examinations and dental questionnaires lasted 25 minutes on average.

### Dental examination

The periodontal examination was conducted by study nurses supported by experienced dentists. Study nurses received a two week intensive training and calibration for these examinations. 250 individuals were examined both by study nurses and dentists. The agreement between both was good: ~95% agreement regarding pocket probing depths between study nurses and dentists on examined sites (N = 6125 out of 6394) within an error range of + −2 mm was present.

Pocket depth (PD) was used as main indicator for the presence of periodontal inflammation. A full-mouth registration for periodontal status was conducted in Heidelberg and a half-mouth registration was carried out in all other study centers. PD was measured on at least two sites per tooth (mesial and mediobuccal) on maxillary and mandible part. For the examination a UNC-PCP15 Color-Coded Probe (Hu-Friedy Europe, Rotterdam/Netherlands) with a black band for each millimeter up to 15 millimeter was used. According to the Community Periodontal Index for Treatment Needs (CPITN) [39] for PD the following definition for periodontitis was used: PD 0-3 mm as no/mild periodontitis, at least one pocket ≥4 mm and <6 mm as moderate and with at least one pocket ≥6 mm as severe periodontitis.

Bleeding on probing (BOP) was measured according to Lang et al. [40] in all study centers except Greifswald. After measuring the PD, the corresponding sites (buccal and mediobuccal) were inspected for the presence or absence of bleeding and noted in an evaluation chart. The absence of BOP can serve as a predictor of periodontal stability [40]. If the percentage of sites with BOP for each person was less than 30% of all probed sites, it was defined as local bleeding only. A percentage of 30% of sites or higher was considered as general BOP [41].

Additional dental status parameters (crowns, implants, dentures, missing teeth, caries and bridges) were recorded for full mouth in all centers, except Berlin, where half mouth assessment was performed. In case of these dental parameters data for Berlin were adjusted to full mouth to allow comparisons with the other centers. Caries was assessed as defined by the International Caries Detection and Assessment System (ICDAS) Code 3 as established decay [42]. Mean numbers were calculated for each dental parameter.

### Anthropometric and socio-economic variables

Age was grouped as 20 to ≤29, 30 to ≤39, 40 to ≤49, 50 to ≤59 and ≥60 years. Body mass index was calculated according to measured values and classified in three different groups <25, 25 to <30 and ≥30. Household income was categorized in two groups: <2000€/month and ≥2000€/month. School education was grouped in two levels. Persons with 13 years of school education (A-Level) and those persons having less than 13 years.

### Lifestyle factors

Alcohol consumption was assessed as frequency of consuming alcoholic drinks and grouped as <2 times/week or never and ≥2 times/week. Smoking was defined as never smokers, ex-smokers and current smokers. Various terms on physical activity (PA) based on “vigorous”, “moderate” and “routes walked” activities were combined and recalculated as metabolic equivalent of task (MET) minutes/week based on the guidelines of the International Physical Activity Questionnaire (IPAQ-Short) http://www.ipaq.ki.se/scoring.pdf grouped into two groups (<1500, ≥1500).

### Blood parameters and pre-existing diseases

Blood parameters were measured with following units: Leukocytes as 1/nl, erythrocytes as 1/pl, mean corpuscular/cell volume (MCV) in fl and HbA1c in mmol/mol. Self-reported diabetes was used as a dichotomous variable (“Yes”/”No”).

### Statistical methods

Data are described descriptively, graphically and by appropriate tables. Univariate associations between lifestyle variables, blood parameters and dental status parameters with PD and BOP were assessed with Kruskal-Wallis-Tests, respectively. To assess joint effects of lifestyle and socioeconomic variables on PD and BOP we used ordinal logistic regression, and binary logistic regression, adjusted for age, sex and center by stratification. Statistical calculations were performed using PROC LOGISTIC in SAS version 9.3 (SAS Institute, Cary, North Carolina).

## Results

The mean age of the participants in this study population was 46.4 years and ranged from 20 years to 77 years. More females (61.4%) than males (38.6%) attended. The sex and age distribution by center is given in Table 2.

**Table 2:** **Demographic characteristics of the study population by center and sex: Number (%) by center, mean age (standard deviation) and age range**

		Augsburg	Berlin	Greifswald	Heidelberg	Total
**Male**	N (%)	19 (43.2)	22 (31.4)	37 (34.6)	42 (46.7)	120 (38.6)
	(s. d.)	56.8 (10.8)	41.4 (13.9)	51.3 (13.7)	43.1 (15.4)	47.5 (14.9)
	Range	(35–70)	(20–67)	(28–77)	(21–69)	(20–77)
**Female**	N (%)	25 (56.8)	48 (68.6)	70 (65.4)	48 (53.3)	191 (61.4)
	(s. d.)	53.7 (11.9)	41.0 (14.5)	48.3 (13.7)	42.6 (14.7)	45.7 (14.5)
	Range	(28–69)	(21–68)	(21–76)	(21–69)	(21–76)
**Total**	N (%)	44 (14.2)	70 (22.5)	107 (34.4)	90 (28.9)	311 (100.0)
	(s. d.)	55.0 (11.4)	41.1 (14.2)	49.3 (13.7)	42.8 (15.0)	46.4 (14.7)
	Range	(28–70)	(20–68)	(21–77)	(21–69)	(20–77)

Table 3 shows the distribution of the maximum PD and the percentage of sites with bleeding on probing (BOP) by study center. Overall, about half of the study population showed no increased PD or only mild form of periodontitis (165/311, 53.1%). The percentage of sites with BOP was 12.4% overall. Individual percentages varied from 0.0% to 82%, and were strongly correlated with maximum PD. In contrast to the sites of BOP the distribution of PD differed significantly between study centers (p = 0.35 and p = 0.001). This can partly be explained by differences in age distribution.

**Table 3:** **Overview on pocket depth (PD) and bleeding on probing (BOP): Number of people on PD by center, mean in percent of BOP sites, range of BOP sites (percent) by center and PD**

	Augsburg		Berlin		Greifswald		Heidelberg		Total	
	N	Mean BOP sites in %), Min-Max in %	N	Mean BOP sites in %), Min-Max in %	N	Mean BOP sites in %), Min-Max in %	N	Mean BOP sites in %), Min-Max in %	N	Mean BOP sites in %), Min-Max in %
**PD Level**										
<4mm	15	3.5	44	10.0	62	NA*	44	4.4	165	6.7
		0.0-15.0		0.0-46.4				0.0-25.0		0.0-46.4
4+5mm	16	9.9	21	22.1	38	NA	26	15.0	101	16.1
		0.0-25.0		0.0-82.1				0.0-40.0		0.0-82.1
≥6mm	13	14.8	5	30.0	7	NA	20	25.3	45	22.3
		0.0-33.3		14.3-42.9				0.0-80.8		0.0-80.8
**Total**	44		70		107		90		311	

*NA Not available in the study center of Greifswald.

In Table 4 the distribution of (i) other dental variables (ii) anthropometric, social and lifestyle factors and (iii) blood parameters by PD level and general BOP status is given. Relations of these variables tend to be stronger with PD level than with BOP values. Individuals with high PD levels had a significantly larger number of teeth with caries, bridges and crowns as individuals with low PD level. Individuals with BOP had a significantly larger number of teeth with crowns than individuals without BOP.

**Table 4:** **Distribution of variable groups by periodontal status (PD and BOP): mean (range) or number (%)**

	PD					BOP			
N	<4 mm	4 + 5 mm	≥6 mm	Total	P ^§^	No	Yes	Total	P ^§^
	166	101	44	311		59	150	209	
**Dental health care variables**									
**No of teeth**	25.8 (6–28)	24.7 (4–28)	22.3 (3–28)	24.9 (3–28)	0.0001	24.6 (13–28)	26.4 (4–28)	25.5 (4–28)	0.05
**No of teeth with caries**	0.10 (0–4)	0.21 (0–4)	0.41 (0–6)	0.18 (0–6)	0.01	0.27 (0–4)	0.23 (0–6)	0.24 (0–6)	0.64
**No of teeth with restorations**	7.45 (0–18)	7.84 (0–16)	7.39 (0–22)	7.57 (0–22)	0.65	7.68 (0–18)	6.41 (0–16)	6.77 (0–18)	0.16
**No of implants**	0.18 (0–4)	0.22 (0–6)	0.11 (0–2)	0.18 (0–6)	0.74	0.27 (0–6)	0.13 (0–6)	0.17 (0–6)	0.45
**No of teeth with bridges**	0.47 (0–7)	1.11 (0–7)	1.50 (0–8)	0.82 (0–8)	<.0001	0.56 (0–7)	0.85 (0–8)	0.77 (0–8)	0.26
**No of teeth with crowns**	2.46 (0–20)	5.19 (0–23)	4.59 (0–18)	3.65 (0–23)	<.0001	3.12 (0–22)	4.06 (0–23)	3.79 (0–23)	0.08
**Lifestyle, anthropometric, health related and sociodemographic variables**									
**BMI**	23.9 (16.6-36.3)	26.1 (17.2-39.6)	27.8 (17.4-43.0)	25.2 (16.6-43.0)	<.0001	24.3 (17.6-36.3)	25.1 (17.2-43.0)	24.9 (17.2-43.0)	0.45
**Diabetes**									
Yes	5 (3.0)	10 (9.9)	4 (9.1)	19 (6.1)	0.06	1 (1.7)	8 (45.3)	9 (4.3)	0.24
No	161 (97.0)	91 (90.1)	40 (90.9)	292 (93.9)		58 (98.3)	142 (94.7)	200 (95.7)	
**Physical activity**									
<1500^&^	52 (31.3)	30 (29.7)	12 (27.3)	94 (30.2)	0.83	16 (17.1)	46 (30.7)	62 (29.7)	0.61
≥1500	114 (68.7)	71 (70.3)	32 (72.7)	217 (69.8)		43 (72.9)	104 (69.3)	147 (70.3)	
**Sex**									
male	61 (36.8)	36 (35.6)	23 (52.3)	120 (38.6)	0.14	19 (50.0)	48 (42.9)	67 (44.7)	0.35
female	105 (63.2)	65 (64.4)	21 (47.7)	191 (61.4)		19 (50.0)	64 (57.1)	83 (55.3)	
**Age**	41.0 (21.0-76.0)	52.1 (20.0-77.0)	54.0 (26.0-69.0)	46.4 (20.0-77.0)	<.0001	42.9 (21.0-68.0)	45.5 (20.0-70.0)	44.8 (20.0-70.0)	0.25
**Household income**									
<2000€	53 (31.9)	36 (35.6)	14 (31.8)	103 (33.1)	0.79	17 (28.8)	53 (35.3)	70 (33.5)	0.37
≥2000€	113 (68.1)	65 (64.4)	30 (68.2)	208 (66.9)		42 (71.2)	97 (64.7)	139 (66.5)	
**School education**									
<13 years	70 (42.2)	65 (64.4)	35 (79.6)	170 (54.7)	<.0001	25 (42.4)	81 (54.0)	106 (50.7)	0.13
13 years	96 (57.8)	36 (35.6)	9 (20.4)	141 (45.3)		34 (57.6)	69 (46.0)	103 (49.3)	
**Smoking**									
Never	98 (59.0)	60 (59.4)	16 (36.4)	174 (55.9)	0.001	32 (54.2)	73 (48.7)	105 (50.2)	0.82
Ex-smoker	36 (21.7)	30 (29.7)	8 (18.2)	74 (23.8)		9 (15.3)	38 (25.3)	47 (22.5)	
Current smoker	32 (19.3)	11 (10.9)	20 (45.4)	63 (20.3)		18 (30.5)	39 (26.0)	57 (27.3)	
**Alcohol**									
<2 times/week	101 (60.8)	65 (64.4)	26 (59.1)	192 (61.7)	0.80	31 (52.5)	92 (61.3)	123 (58.8)	0.25
≥2 times/week	65 (39.2)	36 (35.6)	18 (40.9)	119 (38.3)		28 (47.5)	58 (38.7)	86 (41.2)	
**Blood parameters**									
**Leukocytes**	6.2 (2.9-22.4)	6.4 (3.4-12.4)	7.0 (4.1-11.5)	6.4 (2.9-22.4)	0.04	6.1 (3.1-10.5)	6.6 (2.9-22.4)	6.4 (2.9-22.4)	0.08
**HbA1c**	33.8 (12.6-46.0)	35.8 (22.4-66.0)	40.0 (26.8-60.0)	35.3 (12.6-66.0)	<.0001	37.0 (28.0-46.0)	38.2 (25.0-66.0)	37.3 (25.0-66.0)	0.24
**Erythrocytes**	4.5 (3.4-5.8)	4.5 (3.8-5.5)	4.6 (3.9-5.2)	4.5 (3.4-5.8)	0.89	4.5 (3.4-5.4)	4.5 (3.8-5.6)	4.5 (3.4-5.6)	0.42
**Mean corpuscular/cell volume (MCV)**	87.1 (70.0-96.3)	87.7 (74.0-96.7)	88.8 (81.0-99.9)	87.5 (70.0-99.9)	0.04	87.1 (76.8-94.7)	87.5 (70.0-99.9)	87.4 (70.0-99.9)	0.27

^§^Kruskal-Wallis-Test.

^&^MET (metabolic equivalent of task)/week.

Significantly higher PD levels were also found with increasing body mass index, smoking, lower years of school education and age.

Regarding the blood parameters drawn from blood samples we observed increased values for leukocytes, HbA1c and MCV with increasing PD. No significant associations with BOP were found.

In the multivariable ordinal regression model, adjusted for age, sex and center, BMI and smoking remained significantly associated with PD (see Table 5). Higher BMI (increase by 5, OR = 1.66, 95% CI = 1.25-2.21), and current smoking (OR = 2.44, 95% CI = 1.24-4.80) yield a more severe periodontitis.

**Table 5:** **Results of multivariable ordinal logistic regression on pocket depth (PD), N = 311 and multivariable logistic regression on bleeding on probing (BOP), N = 209: Odds ratio (OR), confidence interval (CI) and p-values**

	PD			BOP		
Effect	OR	95% CI	p	OR	95% CI	p
**Lifestyle and anthropometric factors**						
BMI (increase by 5)	1.66	1.25-2.21	0.0004	1.21	0.81-1.81	0.36
Alcohol (more or equal vs. less than 2 times per week)	0.60	0.34-1.06	0.08	0.61	0.29-1.28	0.19
Physical activity (high vs. low)	1.34	0.79-2.29	0.28	0.90	0.44-1.86	0.78
Smoking never	1			1		
Ex-smoker	1.13	0.61-2.09	0.69	1.94	0.79-4.80	0.15
Current	2.44	1.24-4.80	0.01	1.03	0.46-2.29	0.95
**Socio-economic factors**						
Household income ≥2000€ vs <2000€	0.86	0.51-1.46	0.58	0.77	0.38-1.53	0.45
School (13 years vs. <13 years)	0.61	0.35-1.04	0.07	0.74	0.32-1.70	0.48

Adjusted for age, sex and center.

The logistic regression model for BOP was adjusted for age, sex and center and limited to 209 people. There were no statistically significant associations between the dependent variable BOP and the independent variables.

**Figure 1:**
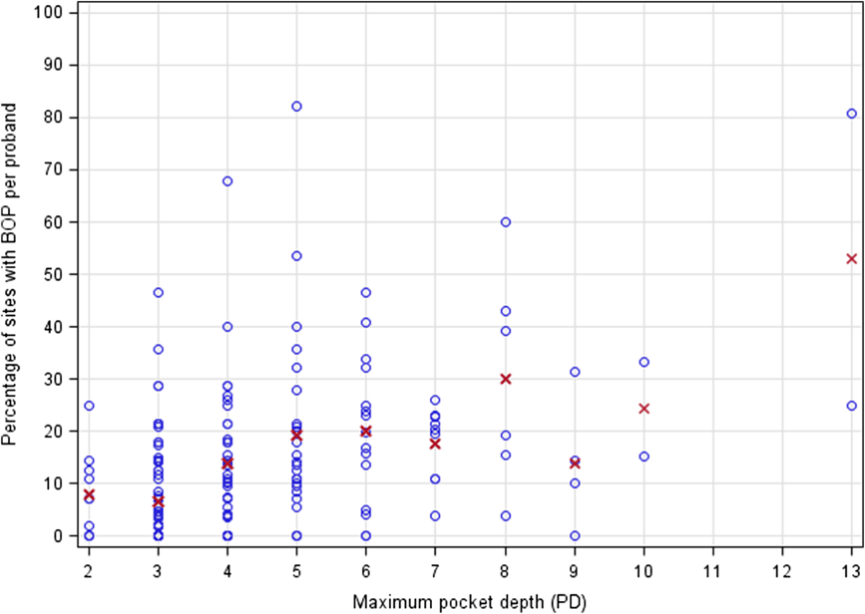
Distribution of maximum pocket depth (PD) with percentages of bleeding on probing (BOP) sites per person in correlation to sites of BOP per patient*. * dots represent individual observations, red x represent mean percentage per pocket depth value.

The two variables PD and BOP were correlated. Figure 1 shows a scatter plot of the individual percentages of sites with BOP against the maximum PD. Mean BOP values were calculated for each value of PD and included in the figure, indicating the correlation between both variables.

## Discussion

This study provides the first data on periodontal parameters in the framework of the planned GNC in Germany and highlights the need for a thorough assessment of oral health conditions in Germany. The prevalence of periodontitis is again found to be high in the German population. This study showed that high BMI and smoking are significantly associated with increasing PD levels.

### (i) Dental parameters

Dental plaque is a shared etiological factor for developing dental caries and periodontitis and it has been discussed that caries and periodontitis are antagonizing [43]. In case of aggressive periodontitis where plaque is not a major etiological factor a lower prevalence of caries lesions can be found compared to patients with chronic periodontitis [44]. However, Kinane et al. [45] were not able to find any patterns of relationship between caries and periodontitis. A possible reason for the effect of caries in our study could be that only cavitated carious lesions were included (ICDAS ≥3). This might represent a subpopulation of people that might neglect dental attendance or dental hygiene. Besides caries also the crowns and bridges showed a significant difference between the three PD groups. This might be as well contributed to the role of plaque and an insufficient ability of proper dental hygiene. In future analyses oral hygiene should be noted at least in a questionnaire.

### (ii) Lifestyle and anthropometric factors

Smoking was positively associated with periodontitis in our study population. However, the use of smoking status in three categories (non-smoking, ex-smoking and current smoking) might have influenced the analyses. Ideally, smoking should be used in further analyses e.g. in amount of pack-years or at least amount of cigarettes smoked. Especially in cases of BOP it has been discussed in recent literature that people have less BOP with higher smoking rates [46]. However, we have not considered this issue further and could not find any associations for BOP with smoking in our analysis. Limitations due to the number of persons for BOP analysis are obvious.

Our findings on BMI are in concordance with already published papers [47]. There was a significant association between BMI and periodontitis and also an increase of BMI with higher ages. Benguigui showed that plaque index and deeper pockets are influenced by BMI level [48]. In particular, the age-specific associations showed the same tendency as in other studies in which a higher BMI was expected with increasing age. However, correlations between age and BMI on the lifestyle could still be present. Issues of nutrition and food intake, less oral hygiene could also have a direct association or indirect relationship with PD, however, we could not test for those factors any further.

### (iii) Blood parameters

Parameters like leukocytes, HbA1c, erythrocytes and mean corpuscular/cell volume (MCV) have been proposed to have an effect on PD and BOP [49]. Patients with periodontitis have a large chronically inflamed wound area, which enable periodontal pathogens or bacterial end-products to get into the blood stream. This can cause systemic changes in blood homeostasis which could favor other chronic systemic diseases.

However, in case of periodontal diseases blood parameters have to be treated with caution. Whereas other variables could have a direct causal link to periodontitis, higher values of laboratory parameters like leukocytes and HbA1c might be due to inflammations from other parts of the body. Hence, interpretation of results based on laboratory parameters is limited. We have therefore not considered laboratory variables in regression analyses. However, Kruskal-Wallis-Test showed only for HbA1c a significant difference between 3 groups with different severity of periodontitis in our study. None of the other laboratory variables were significant when tested for BOP or PD.

Leukocyte counts showed a significant difference within the three periodontitis groups in our study. There are hints in literature supporting the findings of an association of periodontitis with elevated leukocytes but up to date a clear correlation could not be found [49]. A recent study based on three different groups of periodontitis showed similar results on leukocytes as compared to our study population [50].

Signs of anaemia can be associated with chronic inflammatory conditions due to a cytokine mediated depletion of erythropoin [51]. Lainson et al. was one of the first who raised such a question in periodontitis [52]. Erythrocyte parameters like haematocrit, mean corpuscular haemoglobin/volume, haemoglobin and erythrocyte counts were significantly different in patients with aggressive periodontitis [53]. In our study population, however, this can only be partly confirmed by an increase in MCV values within the three groups with different severity of periodontitis.

However, physical activity failed to show any statistical significance and an increase in HbA1c levels in our mostly non diabetic population could be noted. Nevertheless this represents a well known phenomenon [54] thus increased HbA1c values might have an impact on the immune reaction of the periodontium.

All centers followed the standard operating procedures (SOPs) and used standardized questionnaires, which minimizes differences between the centers. Nevertheless differences of trainers- (dentists) and trainees- (study nurses) and inter center methods could vary slightly due to missing calibration prior to the feasibility study. In the present study, differences between the study centers and examiners might be present, although the attempt was made to keep the conditions constant across the centers.

In this study, only a half-mouth assessment for periodontal status was conducted, but the allocation of each side was randomized and was set at two sites per tooth. As a consequence, the findings could not be extended to consider the entire mouth. However, basic dental parameters were investigated full mouth and had to be slightly adapted for Berlin (see Methods section). Some studies performed a half mouth examination, whereas others had full mouth [55]. Studies have shown that in using half-mouth examination there might be some underestimation of disease prevalence [56]. In a recent systematic review half mouth periodontal chart showed a relatively high sensitivity if performed on six sites per tooth [57]. Both methods are convenient and in accordance with clinical methods in the literature, but highly dependent on resources and time constraints [58]. Due to a limited time period, the complete investigation was shortened to some important parts.

We have considered PD and BOP by two regression models independently. In general, there is a correlational link between PD and BOP which we could at least show as the percentages of mean BOP sites increase with deeper pockets. However, no significant differences in BOP were found as they have been associated with the various variables and especially the laboratory parameters [49, 53]. This could have been influenced by the small number of individuals with BOP in this study.

In further research on the prevalence of periodontitis not only PD should be taken into account. Attachment level (AL) is an important factor to define the magnitude and the severity [59] of the disease accurately. Therefore, both parameters are very important in combination and should be considered in further studies/analyses. On the other hand, inter examiner data showed that it was difficult to train study nurses in measuring reliable outcomes in AL. In case of measuring attachment level no reliable data were obtained from study nurses. Because of this it was decided to use maximal pocket depths as a marker for periodontal inflammation and to intensify the clinical training protocol for study nurses. As AL was not generally measured in all centers (only HD) it could not be compared, so only the PD was analyzed commonly.

For future research questions about oral hygiene status have to be included into the study questionnaire because it is likely that there might be confounding effects for tested variables. Ideally in further studies, smoking should be assessed as pack-years, or at least number of cigarettes smoked per day. Socioeconomic variables should be addressed precisely as they often have been discussed in literature to have an effect on periodontal diseases.

## Conclusions

Increased BMI and smoking are associated with periodontitis risk. Dental variables found to be linked with pocket depth are caries lesions, number of bridges and crowns. Further associations of periodontitis with immunological parameters like leukocytes, HbA1c, MCV were found, however, a causal relation cannot be deduced from this study.
